# Environmental and Metabolic Sensors That Control T Cell Biology

**DOI:** 10.3389/fimmu.2015.00099

**Published:** 2015-03-17

**Authors:** George Ramsay, Doreen Cantrell

**Affiliations:** ^1^Division of Cell Signalling and Immunology, University of Dundee, Dundee, UK

**Keywords:** T cell metabolism, glucose uptake, hypoxia, amino acid uptake, leucine and mTOR, aryl hydrocarbon, microbiome

## Abstract

The T lymphocyte response to pathogens is shaped by the microenvironment. Environmental sensors in T cells include the nutrient-sensing serine/threonine kinases, adenosine monophosphate-activated protein kinase and mammalian target of rapamycin complex 1. Other environmental sensors are transcription factors such as hypoxia-inducible factor-1 and the aryl hydrocarbon receptor. The present review explores the molecular basis for the impact of environmental signals on the differentiation of conventional T cell receptor αβ T cells and how the T cell response to immune stimuli can coordinate the T cell response to environmental cues.

## Introduction

T lymphocytes respond to immune stimulation by clonally expanding and differentiating to effector cells that produce the cytokines, chemokines, and cytolytic molecules that mediate adaptive immune responses. This process of T cell differentiation requires the cells to reprogram metabolism to meet the demands caused by the increases in macromolecule biosynthesis that accompany T cell activation (1–4). Naïve peripheral T cells are thus small cells that are metabolically quiescent with low rates of nutrient uptake and protein synthesis. Immune-activated T cells up-regulate glucose, amino acid, and iron uptake in a response that increases cellular energy production to support the biosynthesis of the proteins, DNA, and lipids necessary for cell growth and clonal expansion. Regulation of nutrient uptake is necessary for the differentiation of both naïve CD4 and CD8 T cells into effector and memory sub populations (5–7).

**Figure 1:**
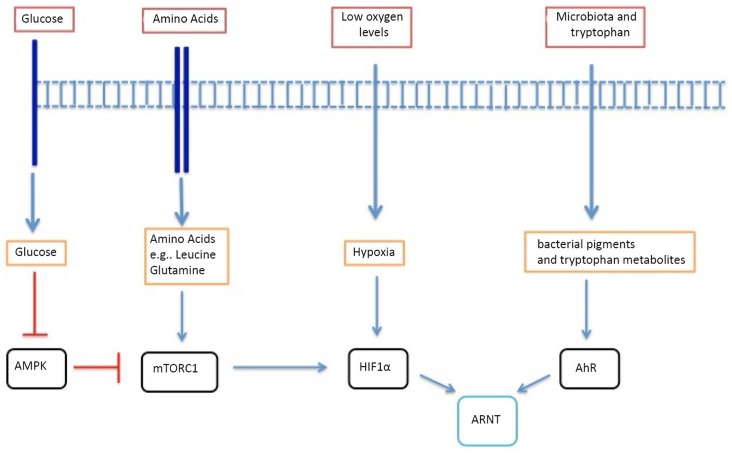
**A summary of the key metabolic and environmental sensors that influence T lymphocyte biology**. mTORc1 is inhibited by AMPK, which is a key glucose sensor. mTORc1 is also influenced by intracellular amino acid levels such as leucine and glutamine. Oxygen tensions are sensed by HIF-1α, which remains under the control of mTORc1. Bacterial virulence factors and metabolites from dietary tryptophan are sensed by the AhR. AhR and HIF-1α have the same dimerizing partner – HIF-1β.

Accordingly, there is the potential for the T cell immune response to be shaped by the T cell nutrient microenvironment. In this context, it is increasingly recognized that antigen and cytokine stimuli coordinate how T cells respond to environmental cues by controlling the expression of nutrient receptors on the T cell membrane and by controlling the expression and function of environmental-sensing transcription factors that control T cell differentiation (5, 8–10) (Figure 1). The present review will explore some recent advances in our understanding of how environmental signals can impact on the differentiation of conventional T cell receptor (TCR)αβ T cells and how the T cell response to immune stimuli can coordinate the T cell response to environmental cues. Specifically, we will review glucose, oxygen, amino acid, and microbiome sensors and their impact on T lymphocyte biology.

## Glucose Sensors and T Cells

Naïve and memory T cells have very low rates of glucose uptake and predominantly produce ATP via oxidative phosphorylation. T cell activation during an adaptive immune response is associated with a rapid up-regulation of the expression of glucose transporters on the T cell membrane and a corresponding increase in glucose uptake. Antigen receptor-activated T cells also change from metabolizing glucose primarily through oxidative phosphorylation to also using the glycolytic pathway (3, 11). T cell immune responses are initiated in response to TCR antigen receptor triggering by cognate peptide/major histocompatibility complexes on the surface of antigen-presenting cells. Moreover, the T cell response to antigen is modulated by multiple adhesion molecules and co-stimulatory molecules and by multiple pro-inflammatory cytokines. These antigen receptor and co-stimulatory molecules coordinate the signaling pathways that control the changes in glucose metabolism that underpin T cell immune responses.

The up-regulation of glucose transporter expression and the glycolytic program of T cells is initiated by antigen and co-stimulatory molecules via the transcription factor c-myc (12). However, the expression of glucose transporters and glycolytic enzymes by antigen-activated T cells can also be sustained by inflammatory cytokines such as interleukin 2 (13–15). One key signaling pathway that sustains glucose metabolism is mediated by the serine kinase mammalian target of rapamycin complex 1 (mTORc1) via regulation of hypoxia-inducible factor-1 (HIF-1) complexes (12, 14, 16). The mTORc1/HIF pathway sustains glucose metabolism in IL-2-activated CD8^+^ T cells by controlling expression of the glucose transporter Glut1 and by regulating expression of hexokinase 2, a key enzyme which phosphorylates glucose to produce glucose-6-phosphate, an essential intermediate in most pathways for glucose metabolism. The mTORc1/HIF pathway also controls expression of rate-limiting glycolytic enzymes in effector T cells such as phosphofructokinase 1, lactate dehydrogenase, and pyruvate kinase M2 (14).

The switch to glycolysis that accompanies T cell activation makes effector T cells dependent on relatively high levels of glucose transporter expression and exogenous glucose in order to sustain their transcriptional program (17, 18). There is not a full understanding of how glucose metabolism controls T cell differentiation. However, one key glucose sensor in T cells is the adenosine monophosphate-activated protein kinase (AMPKα1) (19). This kinase is phosphorylated and activated by liver kinase B1 (LKB1) in response to the increases in cellular AMP:ATP ratios that occur rapidly when metabolically active T cells are glucose deprived (8, 20). The expression of AMPKα1 is not essential for effector T cell proliferation or differentiation but it is necessary for the survival of activated T cells *in vivo* following withdrawal of immune stimulation (8). AMPKα1 null CD8^+^ T cells also show a striking defect in their ability to generate memory cells following pathogen infection. The importance of AMPK for T cells reflects its ability to enforce quiescence to limit energy demands under conditions of energy stress. Hence, a key role for AMPKα1 is to restrain the activity of the mTORc1 (8, 21, 22).

Adenosine monophosphate-activated protein kinase can also stimulate autophagy (23) and in this respect, autophagy has been shown recently to be critical for the formation of CD8 T cell memory (24, 25). The loss of key molecules that control T cell autophagy thus pheno-copies the impact of AMPK deletion on the formation of memory T cells.

Why is it important that activated T cells switch on glycolysis? The glycolytic pathway is a very inefficient way to produce ATP from glucose and it would seem more logical to use oxidative phosphorylation as long as oxygen tensions are sufficient. One explanation is that glycolytic intermediates are used as precursors for nucleotide, amino acid, phospholipid, and triglyceride biosynthesis. It is also noteworthy that non-metabolic functions of glycolytic enzymes have been described (26). For example, it has been described that the glycolytic enzyme GAPDH controls effector T cell production of the cytokine interferon gamma by binding to AU-rich elements within the 3′ UTR of IFN-γ mRNA and hence controlling the translation of this mRNA (18). One other factor to consider is that the glycolytic products lactic acid and succinate can function as “signaling” molecules to control transcriptional responses in macrophages and could well have similar functions in T cells (27, 28).

## Oxygen Sensors and T Cells

One important environmental factor for T cells is the local oxygen (O_2_) tension. The term hypoxia is used to refer to oxygen tensions below the physiological norm and it is now recognized that naïve T lymphocytes recirculate through tissues with quite wide ranges of oxygen saturation. Oxygen tension is thus relatively low in secondary lymphoid tissues such as lymph nodes compared with the arterial bloodstream (5 versus 13%) (29). Other tissues that have comparative hypoxia in healthy hosts include the intestine and skin (30, 31). It is also clear that effector T cells have to function under relatively hypoxic conditions, e.g., at sites of tissue inflammation and within tumor microenvironments where cellular growth rates supersede rates of angiogenesis and oxygen supply.

The main oxygen sensor in T cells is the transcription factor hypoxia-inducible factor alpha (HIF-1α). At atmospheric oxygen tension (21%), HIF-1α is rapidly degraded. This rapid degradation occurs because proline residues of HIF-1α become hydroxylated by prolyl hydroxylases after which HIF-1α is ubiquitinated by the von-Hippel–Lindau (Vhl) E3 ligase complex (32, 33) with the resultant targeting of HIF-1α for degradation. The hydroxylation of HIF-1α requires oxygen as a substrate (34) such that HIF-1α degradation is inhibited when oxygen tension are low. Stabilized, HIF-1α translocates to the nucleus where it dimerizes with HIF-1β (also named the aryl hydrocarbon nuclear translocator). The HIF-1α/HIF-1β heterodimer then binds to hypoxia response elements (HREs) in the promoters of specific genes (29).

**Figure 2:**
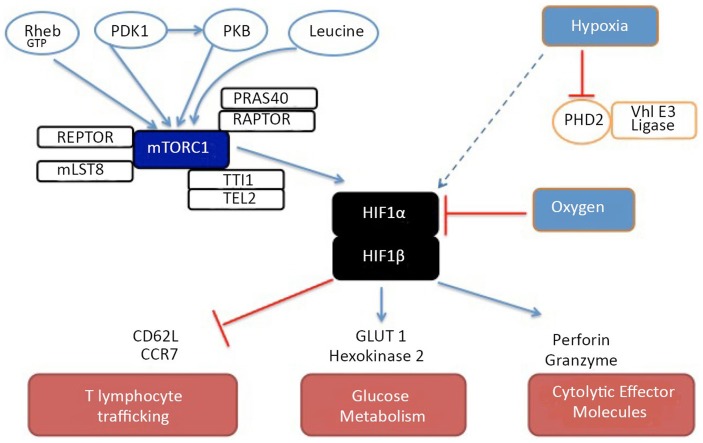
**A model of the regulation and function of HIF complexes in cytotoxic T cells**. Under normoxic conditions, HIF-1α is rapidly degraded. This rapid degradation occurs because proline residues of HIF-1α become hydroxylated by prolyl hydroxylases after which HIF-1α is ubiquitinated by the von-Hippel–Lindau E3 ligase complex. In hypoxia, the hydroxylation of HIF-1α does not occur and the transcription factor is no longer targeted for degradation and HIF-1α protein can thus accumulate within the cell. In effector CD4 and CD8 T cells, high levels of mTORC1 activity promote HIF-1α translation allowing moderate levels of HIF complexes to accumulate even under normoxic conditions. The different regulatory signals that control mTORc1 activity in T cells are also shown as are some of the gene targets whose expression is either promoted (blue arrows) or repressed (red lines) by HIF-1 complexes.

In both CD4^+^ and CD8^+^ T cells, HIF complexes accumulate in effector T cells even under normoxic conditions if these cells have high levels of mTORc1 activity (14, 16). This probably reflects that mTORc1 controls the translation of HIF-1α mRNA. Nevertheless, expression of HIF-1α in effector T cells can also be rapidly enhanced further by exposure to hypoxia (14). The HIF-1 pathway is required to sustain expression of multiple genes encoding proteins that control glycolysis and pyruvate metabolism in effector T cells. The expression of the glucose transporter GLUT1 is HIF controlled in T cells but the ability of HIF-1 to sustain glucose metabolism extends beyond a simple model of HIF-1 regulation of glucose uptake. HIF-1 null effector cytotoxic T lymphocytes (CTL) thus cannot sustain expression of multiple rate-limiting glycolytic enzymes; hexokinase 2, pyruvate kinase 2, phosphofructose kinase, and lactate dehydrogenase. Strikingly, HIF-1 regulates a quite diverse transcriptional program in CTL and in particular controls expression of cytolytic effector molecules such as perforin and granzymes. Indeed, when CTL are switched from normoxic (21%) to hypoxic (1%) oxygen, they substantially increase expression of HIF-1α and perforin (14). These results explain observations that CTL cultured under hypoxic conditions display increased cytotoxic function (Figure 2) (35).

It is also of note that HIF-1-regulated genes in CTL encode chemokine receptors and adhesion molecules. For example, HIF activity regulates the expression of the chemokine receptor CCR7 and the cell adhesion molecule CD62L (L-selectin). These molecules are expressed at high levels in naïve and memory T cells and are essential for lymphocyte transmigration from the blood into secondary lymphoid tissue. In contrast, effector CTL down-regulate CD62L and CCR7 expression as part of the program that redirects effector T cell trafficking away from lymphoid tissue toward sites of inflammation (14). In the absence of HIF transcriptional complexes, effector CTL retain expression of CD62L and CCR7 and they also retain the migratory properties of naïve/memory T cells and preferentially home to secondary lymphoid tissues (14). There is therefore a dominant requirement for HIF-1 for the normal programing of effector CD8 T cell trafficking.

HIF transcriptional complexes, the metabolic sensor of cellular oxygen levels, thus act positively to control expression of glucose transporters and cytolytic effector molecules but have a role to repress expression of critical chemokine and adhesion receptors that regulate T cell trafficking. There are thus fundamental mechanisms mediated by HIF that link oxygen sensing and transcriptional control of CD8 T cell differentiation. There is moreover evidence that HIF signaling is an important regulator of effector CD8 T cell function. Hence, T cell-specific deletion of Vhl, which targets HIF for degradation causes increased pathology in response to chronic viral infection (36) reflecting that HIF signaling seems to limit the terminal differentiation of CD8 T cells. There are parallel data that oxygen sensing acts via HIF to control the differentiation of effector CD4 cells (16). The oxygen tension in the T cell environment will thus have an impact of the T cell differentiation program.

## Regulation of Amino Acid Transport in T Lymphocytes

T cell activation is known to increase rates of amino acid uptake by increasing expression of key amino acid transporters. For example, immune-activated T cells up-regulate expression of glutamine transporters; members of the sodium-dependent neutral amino acid transporter (SNAT) family (5, 7). In the context of glutamine uptake, T cells have an absolute requirement for a large external supply of glutamine and even modest reductions of external glutamine concentrations will negatively impact on T cell proliferation and differentiation (7). Moreover, triggering of the TCR induces expression of the glutamine transporter ASCT2 in T cells and the deletion of ASCT2 suppressed the development of pro-inflammatory CD4 helper 1 (Th1) and Th17 cells *in vivo* in mouse models of autoimmunity highlighting the integration of T cell responses to antigen with changes in T cell glutamine metabolism (7).

Exposure to pathogens and triggering of the TCR in CD8^+^ T cells also induces a striking increase in expression of the System L amino acid transporter complex that comprises a heterodimer of CD98 and the large neutral amino acid transporter (LAT1), encoded by the SLC7A5 gene (5). The regulated expression of System L amino acid transporters is particularly important for T cells as these are responsible for cellular uptake of branched chain and aromatic amino acids such as leucine, isoleucine, tryptophan, and phenylalanine (37). These amino acids are all used for *de novo* protein synthesis. System L transport activity in T cells requires sustained exposure to antigen/pathogen or inflammatory cytokines such as interleukin 2 (IL-2) and is an example of how the T cell response to immune stimuli controls the ability of T cells to respond their environment (5).

The regulated expression of acid transporters is particularly important for T cells as these mediate the cellular uptake of the amino acids required for *de novo* protein synthesis. T cell activation is thus associated with large increases in protein synthesis to support the replication of cellular proteins during cell division and to support the synthesis of the secreted cytokines, chemokines, and effector molecules. For example, there is a requirement for sustained leucine uptake for expression of the metabolic regulator c-myc. Slc7a5-null T cells can thus respond to antigen and cytokines to increase expression of mRNA encoding c-Myc but they do not express c-Myc protein. Accordingly, Slc7a5-null T cells are unable to up-regulate the Myc-controlled “metabolic machinery” required for T cell differentiation (5). For example, Slc7a5-null T cells are unable to increase glucose and glutamine uptake in response to TCR antigen receptor engagement.

One important factor in the context of glutamine metabolism in T cells is that the majority of glutamine taken up by T cells is not used for *de novo* protein synthesis. Instead, it is diverted into metabolic intermediates such as pyruvate and particularly lactate with a small amount being oxidized to carbon dioxide through the citric acid cycle. The conversion of glutamine to lactate occurs via a metabolic process known as glutaminolysis whereby glutamine is metabolized to glutamate which is then converted into α-ketoglutarate. Glutamine metabolized in this way is not only a metabolic fuel but provides substrates for T cells to grow and proliferate (12). For example, α-ketoglutarate can enter an isocitrate dehydrogenase-1 (IDH1)-dependent pathway to synthesize acetyl coenzyme A (AcCoA): a key biosynthetic precursor for fatty-acid synthesis and protein acetylation. Here, it is relevant that AcCoA can be produced from glucose-derived pyruvate but in any cell that has switched to aerobic glycolysis, there is the possibility of a stoichiometric conversion of glucose to lactate which will direct glucose-derived carbon away from the tricarboxylic acid cycle and fatty-acid synthesis. Under these conditions, cells may become completely dependent on IDH1-mediated reductive carboxylation of glutamine-derived α-ketoglutarate for *de novo* lipogenesis. Note, α-ketoglutarate also provides the link between glutamine and the Krebs cycle and here it has been noted that T cell activation is associated with increased expression of key enzymes such as involved in glutamine/α-ketoglutarate metabolism notably glutamate dehydrogenase (GDH), glutamic-oxaloacetic transaminase (GOT), and glutamic-pyruvic transaminase (GPT) (38).

It is also noteworthy that mutations in (IDH1) and IDH2 have been found in acute myeloid leukemias (AMLs). These mutant IDH enzymes have a gain of function activity to catalyze the NADPH-dependent reduction of α-ketoglutarate to 2-hydroxyglutarate (2HG). This latter metabolite can act as a competitive inhibitor of histone lysine demethylases and hence control histone demethylation and chromatin structure (39). These observations reveal a clear link between metabolic processes and the cellular epigenome and afford another insight as to how metabolic signaling can impact on cellular transcriptional programs.

## Mammalian Target of Rapamycin: An Amino Acid Sensor in T Cells

One of the main reasons that it is critical that T cells tightly regulate expression of System L amino acid transporters and glutamine transporters is because the intracellular supply of leucine and glutamine regulates the activity of the serine/threonine kinase complex mTORc1 (2, 40–42). The leucine-sensing pathway that activates mTORc1 activity is initiated within lysosomes and involves amino acid-dependent activation of the guanine nucleotide exchange activity of the Ragulator complex. This results in the accumulation of active GTP-bound RagA GTPases, which then recruits mTORc1 to the lysosomal surface where it interacts with the small GTPase Rheb, a potent stimulator of mTORc1 kinase activity in T cells (43, 44). The full details of how T cells sense intracellular leucine are not known but it importance and the importance of mTORc1 for effector T cell differentiation has been well documented (2, 5, 45). The molecular basis for the immunosuppressive actions of mTORC1 inhibitors such as rapamycin is multifaceted. In CD4 T cells, rapamycin inhibition of mTORC1 prevents the differentiation of Th1 and Th17 but not Th2 cells. Rapamycin also causes retention or re-expression of FoxP3 in antigen receptor-activated CD4 T cells and promotes the production of “regulatory” T lymphocytes (2, 45). Inhibition of mTORC1 with rapamycin can also shape CD8 T cell responses to antigen: rapamycin treatment thus prevents effector T cell generation and promotes memory T cell formation (46).

The molecular basis for the importance of mTORc1 for T cell function is not fully understood and is not the focus of the present review. However, as discussed above, one key function for mTORc1 is to control expression of HIF-1 transcription factor complexes which then regulate expression of a diverse array of genes including glucose transporters, glycolytic enzymes, adhesion molecules, and cytolytic effector molecules (14, 16). mTORc1-controlled signaling pathways can also control expression of the sterol regulatory element-binding proteins (SREBP1 and SREBP2); transcription factors that are required for the expression of lipogenic enzymes (47). mTOR has thus been linked to the control of lipid biosynthesis in many cells. The relevance of the SREBP pathway in T lymphocyte biology was suggested when it was demonstrated that phospholipid-dependent kinase 1 (PDK1), which controls mTORc1 activity in CD8 T cells (14), controls the transcription of SREBP gene targets such as hydroxysteroid (17β) dehydrogenase 7, fatty-acid desaturase 2, farnesyl diphosphate synthetase, and stearoyl-coenzyme A desaturase 1 (13). Moreover, SREBP null CD8 T cells cannot metabolically reprogram to glycolysis and show attenuated clonal expansion in response to a pathogen challenge (48). In this context, there is an increasing awareness that the regulation of *de novo* fatty-acid synthesis and fatty-acid catabolism is important for peripheral T cell differentiation (49–51).

## Microbiome Sensors and T Cells

All mammals are colonized with diverse commensal microbes especially at barrier sites such as the skin and the gastrointestinal tract and it is now recognized that these resident bacteria have a key role in controlling immune cell homeostasis and can modulate the function of T cells in adaptive immune responses (52, 53). Importantly, commensal bacteria constantly trigger responses in gut epithelial cells via host microbial pattern recognition receptors particularly Toll-like receptors (TLRs). These signals are essential under normal non-pathogenic steady-state conditions to maintain intestinal epithelial homeostasis (54). Commensal microbes are also essential for controlling the function of lymphocytes at barrier sites. For example, germ-free mice have smaller Peyer’s patches and fewer T and B cells in the intestine than mice with a normal microbial flora. It has also been shown that the differentiation of effector T cell populations can be influenced by the gut microflora. For example, segmental filamentous bacteria (SFB) that adhere tightly to the epithelial layer of the intestine have been shown to drive the differentiation of mucosal Th17 cells (55–58). As well, the maintenance of regulatory T lymphocytes in the gut can be modulated by intestinal bacterial colonization (59).

The molecular mechanisms whereby the bacterial environment controls T cells are complex and a full analysis is beyond the scope of the present review. One element is that bacterial recognition by pattern recognition receptors expressed by epithelial cells, macrophages, and dendritic cells will govern the production of cytokines and chemokines that can direct T cell fate (60, 61). Hence, the nature of the pathogen insult/challenge to the innate immune system, and the resultant cytokine milieu plays a key role in regulating peripheral T cell differentiation. One more direct pathway that interprets the microbial environment for T cells is mediated by the aryl hydrocarbon receptor (AhR), a transcription factor that has been extensively studies in the context of cellular responses to environmental toxins. Ligands for the AhR include dioxins and in the context of toxin binding the AhR translocates to the nucleus to form a complex with the AhR nuclear translocator (ARNT) or HIF-1β. This complex then regulates expression of members of the cytochrome P450 family such as CYP1A1, which function as xenobiotic metabolizing monooxygenases (10). In the context of the influence environmental cues have of lymphocyte population, it is of interest that both HIF-1α and AhR have the same dimerizing partner HIF-1β/ARNT. This suggests a potential for cross talk between the hypoxia-induced factors and the AhR pathways.

The AhR has an important physiological role in T cell immune responses and can regulate the differentiation of effector CD4 T cells that make the pro-inflammatory cytokines IL-17 and IL-22 (62). The AhR also controls the development of innate lymphoid cells (ILCs) at barrier sites such as the intestinal mucosa and the skin (63). The direct gene targets for the AhR that explain how it controls T cell differentiation are not known but could be the cytokine gene promoters themselves.

The AhR was first studied in the context of environmental toxins such as dioxins [e.g., d2,3,7,8-tetrachlorodibenzo-*p*-dioxin (TCDD) ligands]. However, it is the identity of physiologically relevant AhR ligands that has captivated the immunological community. One key discovery was that exposure of tryptophan to ultraviolet light from solar irradiation (in particular UVB) results in the production of a potent AhR ligand 6-formylindolo(3,2-b)carbazole (FICZ) (10). Moreover, AhR null mice have a 90% reduction of TCRγδ^+^ skin T cells. (64). In addition, activation of the AhR dampens the severity of immune-mediated skin inflammation (65).

It has been proposed that AhR activity in gut lymphocytes can be regulated by the metabolism of nutritional components notably tryptophan catabolites produced by diets rich in cruciferous vegetables (66). One product of tryptophan metabolism that functions as an AhR ligand is kynurenine (67, 68). In this respect, high levels of kynurenine production by human tumors have been proposed to suppress antitumor immune responses and hence promote tumor-cell survival (67). The generation of kynurenine from tryptophan metabolism occurs via the enzymes indoleamine 2,3-dioxygenase (IDO) and tryptophan 2,3-dioxygenase (TDO).

The production of AhR ligands from tryptophan metabolites could explain how the diet can shape gut immune responses. The AhR is thus important for the development and maintenance of multiple subpopulations of gut lymphocytes. (69). However, one consideration is that diet alterations might change the bacterial milieu and indirectly regulate gut lymphocytes by controlling the diversity of gut commensal bacteria. In this respect, there is an increasing recognition that the bacterial microbiome can produce ligands for the AhR notably tryptophan metabolites. The AhR can thus act as a link between the host immune system and the commensal microbiota. There is also recent evidence that AhR is able to sense pathogenic microbes. Bacterial-pigmented virulence factors, namely phenazines from *Pseudomonas aeruginosa* and the naphthoquinone phthiocol from *Mycobacterium tuberculosis*, are thus strong AhR ligands. Importantly, there is evidence that AhR null mice are more prone to infections with these organisms (70).

## Conclusion

T cell responses to pathogens can be shaped by the localized microenvironment. T cell differentiation can be regulated by the bacterial environment because many of the cytokines and chemokines produced when pattern recognition receptors in cells of the innate immune system are triggered by bacterial products and subsequently control the adaptive immune response by acting on T cells. What is now emerging is that the bacterial environment can more directly regulate T cell responses. For example, T cells can be directly regulated by bacterial products that activate the AhR transcriptional pathway.

One other advance in our understanding of T cell biology is how the oxygen and nutrient environment can shape T cell differentiation. Moreover, that T cells are not just passive in the way they interact with their environment. T cells can thus control how they respond to the environment by restricting expression of high levels of nutrient receptors and environmental-sensing transcription factors to immune-activated T cells. The value of these insights is that they inform novel strategies to therapeutically manipulate T cell immune responses. For example, metformin, a drug that inhibits the respiratory chain complex I can promote the development of memory CD8 T cell-mediated immune responses arguing that it might be useful tool in vaccine development (71).

Tactics that promote signaling via the oxygen sensor HIF-1α have also been shown to promote the production of effector CD8 T cells, which could be important for strategies aimed at the clearance of viruses and tumors. In a similar context, strategies that activate the AhR have been shown to reduce inflammation in autoimmune-mediated skin conditions. Metabolic diseases such as obesity and diabetes could also be managed by modulation of the immune system. Recent discoveries have identified that innate lymphoid cell 2 subset (ILC2s) dysregulation is a conserved feature of obesity and manipulation of the IL-33/ILC2 axis may lead to therapies for this disease (72). The field of immunotherapy is entering a new era where targeting of pathways that influence the way T cells sense their environment may yield immune-modulatory drugs that are as valuable as those that currently target T cell-specific antigen and cytokine receptor signaling molecules.

## Conflict of Interest Statement

The authors declare that the research was conducted in the absence of any commercial or financial relationships that could be construed as a potential conflict of interest.
